# Academic Outcomes in High-School Students after a Concussion: A Retrospective Population-Based Analysis

**DOI:** 10.1371/journal.pone.0165116

**Published:** 2016-10-20

**Authors:** Kelly Russell, Michael G. Hutchison, Erin Selci, Jeff Leiter, Daniel Chateau, Michael J. Ellis

**Affiliations:** 1 Department of Pediatrics and Child Health, University of Manitoba, Winnipeg, Canada; 2 Children’s Hospital Research Institute of Manitoba, Winnipeg, Canada; 3 Canada North Concussion Network, Winnipeg, Canada; 4 Faculty of Kinesiology and Physical Education, University of Toronto, Ontario, Canada; 5 Department of Surgery, University of Manitoba, Winnipeg, Canada; 6 Manitoba Center for Health Policy, University of Manitoba, Winnipeg, Canada; 7 Department of Surgery, Section of Neurosurgery, University of Manitoba, Winnipeg, Canada; Kessler Foundation, UNITED STATES

## Abstract

**Background:**

Many concussion symptoms, such as headaches, vision problems, or difficulty remembering or concentrating may deleteriously affect school functioning. Our objective was to determine if academic performance was lower in the academic calendar year that students sustain a concussion compared to the previous year when they did not sustain a concussion.

**Methods:**

Using Manitoba Health and Manitoba Education data, we conducted a population-based, controlled before-after study from 2005–2006 to 2010–2011 academic years. Grade 9–12 students with an ICD9/10 code for concussion were matched to non-concussed controls. Overall changes in grade point average (GPA) were compared for the academic year prior to the concussion to the academic year the concussion occurred (or could have occurred among non-concussed matched students).

**Results:**

Overall, 8240 students (1709 concussed, 6531 non-concussed students) were included. Both concussed and non-concussed students exhibited a lower overall GPA from one year to the next. Having sustained a concussion resulted in a -0.90% (95% CI: -1.88, 0.08) reduction in GPA. Over the same period, non-concussed matched students’ GPA reduced by -0.57% (95% CI: -1.32, 0.19). Students who sustained a concussion during high school were just as likely to graduate within four years as their non-concussed peers (ORadj: 0.84; 95% CI: 0.73, 1.02).

**Conclusions:**

We found that, at a population level, a concussion had minimal long-term effects on academic performance during high school. While academic accommodations and Return-to-Learn programs are an important component of pediatric concussion management, research is needed to identify risk factors for poor academic performance after a concussion and who should receive these programs.

## Introduction

Concussion is a form of traumatic brain injury (TBI) defined as a ‘complex pathophysiological process affecting the brain, induced by biomechanical forces’ [1]. Diagnosis is largely based on the presence of physical, cognitive, sleep, and emotional symptoms [1,2] that reflect a disturbance of brain function rather than a structural injury [3,4]. The most commonly reported symptoms include headache, dizziness, loss of balance, confusion, and disturbances in vision and concentration [5–7]. While the majority of symptoms will spontaneously resolve within 7–10 days among adults [8,9], recovery for children and adolescents appears to be longer [10,11]. Overall, 29–73% of children and adolescents will develop prolonged symptoms or be diagnosed with post-concussion syndrome (PCS) [12–14].

Initial concussion management largely focuses on physical and cognitive rest [1]. Return-to-play (RTP) guidelines assist concussed athletes to safely return to sport through a graduated protocol of increasing levels of physical exertion [7,15]. Current RTP guidelines focus almost exclusively on the development of physical symptoms during increased levels of physical activity [16,17]. Cognitive rest refers to avoiding tasks that require attention or concentration, such as completing schoolwork, reading, studying, or using a computer until symptoms resolve [18]. In comparison to RTP, returning to the classroom has received less attention [16]. Studies suggest that symptoms such as headaches, fatigue, impaired concentration, and slowed processing speed may impair academic performance [19,20], therefore students with concussion may be at risk of falling behind in the classroom. Together, these issues could result in students trying to work harder in an attempt to make up missed school work, which may exacerbate concussion symptoms.

Concussion can lead to temporary alterations in neurocognitive functioning [21,22] but it is unclear how this injury impacts school performance. College athletes who suffered multiple concussions had significantly lower grade-point averages (GPAs) than athletes with no concussions [23] but results were not adjusted for potential covariates or pre-concussion GPA. A randomized controlled trial found that strict rest of 5 days (no school, work, or physical activity) versus 1–2 days resulted in slower symptom recovery; however, academic outcomes were not assessed [24]. Concussed high school students self-reported significantly more adverse academic effects such as difficulties studying, paying attention, or taking notes compared with younger students [25]. In this study, both parents and students reported that their grades were negatively affected and students reported increased difficulties in math and science. Despite this insight, there are limited studies examining the effect of concussion on objective measures of academic performance.

We explored the relationship between concussion and academic performance among Grade 9–12 students in the province of Manitoba, Canada using population-based data. The primary objective was to determine if academic performance was lower in the academic calendar year that students sustain a concussion compared to the previous year when they did not sustain a concussion. Secondary objectives included determining if the relationship between concussion and academic performance differed by subject matter (math, sciences, social studies, English, foreign languages) and if students with a concussion were just as likely to graduate from high school compared with students with no history of concussion.

## Methods

### Study Design

We conducted a population-based retrospective controlled before-after study. The ‘pre concussion’ period was defined as the school year prior to sustaining a concussion and the ‘post concussion’ period was the end of the school year when the concussion occurred.

### Data Registries

All data were accessed through the Manitoba Center for Health Policy (MCHP), which contains population-based de-identified health, education, social, and judicial data from administrative records of Manitobans. MCPH’s goal is to conduct timely population-based research on health services, population and public health and the social determinants of health. For this project, databases were accessed from 2005 Manitoba Health’s Administrative Health Database and is the provincial gold standard for demographic information. Concussed adolescents were identified from Manitoba Health’s Medical Services Database or the Hospital Discharge Abstracts Database. Both databases include the first three alpha-numeric ICD codes. In the Medical Services Database, ICD-9 code 850 (concussion) was included. The Hospital Discharge Abstracts Database was accessed to identify hospitalized concussions using ICD-10. Concussion was captured using the S06 code (all intracranial injuries).

Academic outcomes were extracted from Manitoba Education’s Enrollment, Marks, and Assessments Database. This includes enrollment status, courses, teacher assigned end of year final grades (0–100%), and graduation status for Manitoba students who are registered in public school, private schools, or are homeschooled. Students attending school on reserves are not captured. Data were accessed from the 2005–2006 until 2010–2011 academic years. This allowed us to determine academic performance at the end of the academic year that the concussion was sustained and for more students to reach the age of graduation. Data for subsequent academic years were not available at the start of this study.

### Subjects

Students were included if they 1) received a physician-diagnosed concussion in the 2005–2006 to 2010–2011 academic calendar years, 2) registered in grades 9 through 12 at a Manitoba public high school or private high school, and 3) had teacher assigned grades for the school years before and after they sustained their concussion. For students enrolled in full year courses, students were excluded if the concussion occurred in May or June. For students who attended semestered schools, the concussion could not have occurred in the last month of the semester. Because students who sustain a concussion near the end of their course may have difficulties returning to classes, teachers may assign their pre-concussion grade as their final grade in lieu of traditional testing or exams. This would attenuate the findings since the most affected students would appear to have no change in academic performance. Due to the abbreviated schedule, summer school was excluded.

A matched group of non-concussed students was included to ensure that any observed changes were due to concussion and not a naturally occurring phenomenon of grade changes over time. Matched students were those who never had a physician-diagnosed concussion after the age of 10 years. For every concussed student, up to five matched students were selected. Matching criteria included attending the same school, in the same academic year, and enrolled in the same school subjects at the same level (university track on non-university track) as the concussed student.

### Outcomes

Students are assigned a grade between 0–100% for each class (in the rare event they receive a letter grade, Manitoba Education converts this to a percentage grade prior to being entered in the database). The primary outcome was change in academic performance as measured by percent change in overall GPA. The GPA was calculated from end of year teacher assigned grades (sum of all grades divided by the number of courses) and represented as a number between 0% and 100%. Each course was equally weighted. Only core courses were included: math, English, sciences, social studies, and foreign languages. Change in GPA was defined as the difference between the end of year GPA from the year prior to the concussion and the end of year GPA in the same school year as the concussion. Secondary outcomes were course specific change in GPA and the proportion of students who graduated high school within four years with at least the required 28 credits.

### Covariates

The following variables were also extracted from the Administrative Health Database: sex, socioeconomic status (income quintiles), urban versus rural school (Winnipeg or Brandon versus other), public versus private school, academic year, grade, and level of class (university track versus non-university track).

### Ethics

Ethical approval was obtained by the University of Manitoba's Research Ethics Board. Consent was not obtained because all patient records and information are de-identified when entered into Manitoba Health and Manitoba Education database. All analyses were conducted using de-identified data.

### Data Analysis

#### Data Linkage

Within the datasets, each individual is anonymized and assigned a unique identifier based on their scrambled Personal Health Information Number. This identifier links individuals in the Manitoba Health and Manitoba Education datasets to follow individuals through time.

#### Analysis

Courses were grouped into subject areas according to Manitoba Education Subject Table Handbook (e.g., math included accounting, applied math, advanced math, essential math, pre-calculus, calculus, and transitional math) [26]. Multilevel regression models (i.e., mixed models or hierarchical linear models) were performed on all outcomes to determine the impact of a concussion on school performance. These models take into account the matched groups of students (up to 5:1 matches per case) and produce unbiased estimates of effects [27]. A significant interaction between time (pre versus post) and group (concussed versus non-concussed) reflects that the change in grades for concussed students was different than the change in grades for non-concussed students. In other words, these results would indicate that a concussion had an impact on school performance. Estimate statements were included in these models to evaluate the effect of group at each time point, and the effect of time within each group. Models without covariates were run to calculate unadjusted GPAs. For adjusted GPAs, models were run with sex, grade, academic year, SES, urban vs rural school, and level of class included as covariates. Change in GPAs were expressed as a positive or negative percent change in GPA with 95% confidence intervals (e.g., a 5% reduction in GPA would represent pre-concussion GPA of 80% and a post-concussion GPA of 75%). SAS Version 9.3 (TS1M0)^®^ was used for data management, programming, and analyses[28].

## Results

### Subjects

There were 2784 students between ages 14–18 who sustained a concussion; however, 18 were not enrolled in school. An additional 175 students were excluded because they were not enrolled in any core courses. Nine students were excluded because their concussion occurred at the end of the term. Of the remaining 2582 students, 1165 students were excluded because they did not have academic outcomes before their concussion (n = 855, e.g., they were concussed in Grade 9 so their pre-concussion marks were from Grade 8) or academic outcomes after their concussion (n = 310, e.g., moved mid school year). For the remaining 1417 cases, 3867 non-concussed students were identified for the matched analysis to 1093 concussed students. No matches were identified for 324 concussed students. The baseline characteristics of the students who did and did not sustain a concussion are presented in Table 1. There were 1709 concussed and 6531 non-concussed matched students who could have graduated within the four years.

**Table 1:** Crude mean GPA for concussed and non-concussed (matched) students by covariates.

		Concussion Students (N = 1417) (mean GPA%, 95% CI)	No Concussion (matched) (N = 3867) (mean GPA%, 95% CI)
Pre-Concussion	Overall	69.59% (68.91,70.28)	72.99% (72.63,73.35)
	Male	67.82% (66.98, 68.64)	71.19% (70.70. 71.67)
	Female	72.93% (71.77, 74.09)	74.90% (74.40, 75.46)
	Rural	69.53% (68.44, 70.61)	73.06% (72.50, 73.62)
	Urban	69.64% (68.75, 70.52)	72.94% (72.46, 73.41)
	SES Lowest	66.53% (64.17, 64.89)	71.83% (70.51, 73.16)
	SES Low	67.77% (66.01, 69.54)	70.80% (69.91, 71.69)
	SES Middle	67.88% (66.44, 69.31)	72.52% (71.73, 73.30)
	SES High	70.65% (69.22, 72.89)	73.25% (72.50, 73.99)
	SES Highest	72.27% (71.08, 73.47)	74.82% (74.19, 75.33)
	Grade 9	71.14% (69.94, 72.34)	74.57% (73.97, 75.17)
	Grade 10	69.32% (68.13, 70.51)	72.46% (71.85, 73.07)
	Grade 11	68.75% (67.60, 69.91)	71.48% (70.81. 72.16)
	Grade 12	56.68% (50.39, 62.97)	N/A
	Year 2005	68.22% (66.74, 69.82)	73.07% (72.29, 73.85)
	Year 2006	68.94% (67.28, 70.61)	73.05% (72.23, 73.87)
	Year 2007	70.30% (68.78, 71.81)	73.29% (72.53, 74.05)
	Year 2008	70.56% (69.13 72.00)	72.82% (72.00, 73.63)
	Year 2009	69.93% (68.43, 71.43)	72.67% (71.77, 73.57)
	University Track	69.94% (69.27, 70.61)	73.07% (62.07, 69.19)
	Non-University Track	55.02% (47.75, 62.28)	65.63% (62.08, 69.19)
	Year 2009	68.07% (66.59, 69.55)	71.78% (70.91, 72.64)
	Year 2010	67.39% (65.66, 65.66)	71.87% (70.89, 72.85)
	University Track	68.84% (68.15, 69.54)	71.70% (71.30, 72.11)
	Non-University Track	52.83% (48.81, 56.86)	68.75% (66.32, 71.18)

N/A: Too few students and data could not be presented

### Overall Change in GPA

Concussed students had significantly lower GPAs than non-concussed matched students, even before the concussion event (p<0.0001). At both pre-concussion and post-concussion time points, concussed students had lower overall adjusted GPAs (pre: 2.24%; post: 2.57%) than non-concussed students. Both concussion and non-concussed matched students’ had lower adjusted GPA over time (concussion: 0.90%; non-concussion: 0.56%) (Table 2), that is, both groups of students had a lower GPA from one year to the next.

**Table 2:** Crude and adjusted mean GPA for concussed and non-concussed (matched) students.

	Pre-Concussion Time Period (mean GPA%, 95% CI)	Post-Concussion Time Period (mean GPA%, 95% CI)
Concussion Students (N = 1417)	Crude: 69.59% (68.91,70.28)	Crude: 67.90% (67.18,68.62)
	Adjusted: 65.03% (63.89,66.18)	Adjusted: 64.13% (63.00, 65.25)
No Concussion (matched) Students (N = 3867)	Crude: 72.99% (72.63,73.35)	Crude: 71.59% (71.20,71.99)
	Adjusted: 67.27% (66.18,68.35)	Adjusted: 66.70% (65.64,67.76)

There was no significant difference in change in GPA after the index date of the concussion occurred (time-concussion status interaction term: p = 0.46). Among concussed students, overall adjusted GPA did not significantly decrease after concussion (0.90%; 95% CI: -1.88, 0.08) and this was observed for the change in overall adjusted GPA among non-concussed students (-0.56; 95% CI: -1.32, 0.19). However, those who went on to sustain a concussion did have significantly lower adjusted GPA than those with no concussion, even prior to the concussion event (2.40; 95% CI: -2.89, -1.92). Students who were male or in the lowest four socioeconomic status quintiles had significantly lower GPA from one year to the next, regardless of concussion status (Table 3). Compared with Grade 9 overall adjusted GPA, students in Grades 10–12 had significantly lower GPAs. Students enrolled in courses required for university acceptance had significantly higher overall adjusted GPAs than students not taking courses required for future university studies.

**Table 3:** Adjusted percent change in overall grade point average.

Variable	% Change in Grade (95% CI)	P-Value
Concussed vs Non-Concussed (matched) Students	-2.40 (-2.89, -1.92)	<0.001
Pre vs Post Concussion Year among Concussed Students	-0.90 (-1.88, 0.08)	0.07
Pre vs Post Concussion Year among Non-Concussed (matched) Students	-0.56 (-1.32, 0.19)	0.142
Male vs Female	-4.20 (-4.65, -3.75)	<0.001
Urban vs Rural School	-0.14 (-0.95, 0.67)	0.7381
Socioeconomic Status (Quintiles)		
Lowest vs Highest	-3.22 (-4.10, -2.34)	<0.001
Low vs Highest	-4.06 (-4.81, -3.30)	<0.001
Middle vs Highest	-2.94 (-3.64, -2.23)	<0.001
High vs Highest	-1.09 (-1.74, -0.44)	0.0010
Grades		
10 vs 9	-1.90 (-2.68, -1.13)	<0.001
11 vs 9	-2.72 (-3.80, -1.65)	<0.001
12 vs 9	-2.20 (-3.70, -0.70)	0.0040
Academic Year		
2006 vs 2005	0.77 (-0.12, 1.66)	0.0895
2007 vs 2005	1.10 (0.004, 2.20)	0.0491
2008 vs 2005	0.57 (-0.61, 1.76)	0.3419
2009 vs 2005	0.82 (-0.49, 2.13)	0.2193
2010 vs 2005	1.33 (-0.33, 2.99)	0.1154
University vs Non-University Track Courses	8.36 (6.92, 9.81)	<0.001

### Course Specific Changes in GPA

Course specific grades were compared before and after the index date of the concussion. There was no significant change in adjusted GPAs for math or English regardless of concussion status (Table 4). Non-concussed matched students had significantly lower adjusted GPA in science (p = 0.036), while concussed students had significantly lower adjusted social studies GPA (p = 0.017) but higher adjusted GPA in foreign languages (p = 0.003).

**Table 4:** Course-specific adjusted changes in GPA among students with and without a concussion (matched students).

	Math (%, 95% CI)	English (%, 95% CI)	Science (%, 95% CI)	Social Studies (%, 95% CI)	Forgein Language (%, 95% CI)
Concussion—Change in Grades	N = 1374	N = 1390	N = 1308	N = 794	N = 84
	-0.42 (-1.58,0.74)	-0.15 (-1.28,0.98)	-0.92 (-2.18,0.34)	-2.20 (-4.01,-0.39)	7.38 (2.57, 12.20)
No Concussion(matched students)—Change in Grades	N = 3857	N = 3637	N = 3774	N = 2269	N = 184
	-0.55 (-1.39,0.29)	-0.44 (-1.27,0.4)	-1.25(-2.41,-0.08)	-1.70 (-5.09,1.68)	2.13 (-1.98,6.25)

All change in grades were adjusted for sex, socioeconomic status (income quintiles), urban or rural school, grade level, academic year the class was taken in, and level of course (university track or not university track).

### Graduating On Time

Overall, 2582 concussed students were eligible to graduate from high school within four years and 84.7% graduated on time. In the same time period, 6531 non-concussed matched students were eligible to graduate on time and 87.0% did so. Students who sustained a concussion during high school had a 0.84 adjusted odds (95% CI: 0.73, 1.02) of graduating high school in four years compared to those with no concussion, but this association was not statistically significant (p = 0.09).

## Discussion

The richness of the Manitoba Center for Health Policy databases provided a unique opportunity to longitudinally examine objective measures of academic performance following concussion. Using population-based data, we found that Manitoba students in Grades 9–12 did not have lower overall GPAs at the end of the academic year in which they sustained a concussion compared to non-concussed students. There was a statistically significant 1.3% reduction in sciences grades among non-concussed students and a 2.2% reduction in social studies grades, and a 7.4% increase in foreign language grades among students with a concussion. In addition, having a concussion during high school did not affect the odds of graduating on time. We did find that males and students of lower socioeconomic status did have significantly lower adjusted GPAs, regardless of concussion status. A recent meta-analysis of more than one million students found that females achieve higher grades in all subjects than their male counterparts [29]. In addition, American researchers have found that poverty was also associated with decreased academic performance [30].

Some students may require temporary short-term or long-term academic accommodations and Return-to-Learn (RTL) programs could be an effective way to manage these students. However, our population-based findings indicate that there are minimal objective academic consequences of sustaining a concussion when examining grades at the end of the academic year. This may aid to alleviate some of parents’ potential fears about negative long-term consequences of concussion associated with sport participation [14].

Other research has found that students suffer academically following one concussion [23,25] or repeat concussions [23,31]. Our study is the first one to use population-based data of students with a physician-diagnosed concussion and examine changes in course-specific grades. Although concussed students exhibited a 2% drop in social studies, this likely does not represent a clinically or academically meaningful change since Grade 12 social studies is not required to graduate or obtain admission to one of the two provincial universities [32].

Graduated Return-to-Play protocols have been the focus of most published expert position statements [15,16,33] and limited research attention has been paid to the effects of concussion on objective measures of academics. Multi-disciplinary consensus statements highlight the role of RTL programs following concussion [34–36], but there is limited empirical evidence to guide the implementation of these programs. Additional research is needed to identify students who may require or benefit from individualized patient-tailored school accommodations that may facilitate safe and expedient return to full school participation. Such efforts would likely require strong collaborations with the medical team, school administrators, teachers, and parents.

### Limitations

This study has several limitations. There may be selection bias because students who were homeschooled or enrolled in First Nation schools are excluded. However, only 0.9% of Manitoba students were homeschooled in Grades 9–12 and 1.3% were enrolled in a First Nations school [37]. The Medical Services Database (ICD-9) and Hospital Discharge Abstracts (ICD-10) databases record the first three alpha-digits only. For ICD-10 codes, intracranial injuries (TBI) are captured in the first three alpha-digits, including concussions. The ICD-9 code for concussion is captured in the first three alpha-digits and most concussions were identified using the Medical Services Database as this injury rarely requires hospitalization [38,39]. Therefore, it is unlikely that we captured many intracranial injures that were not concussions.

There may be misclassification by grades, as there is some subjectivity in teacher assigned grades. One teacher may assign 90% for the same quality of work that their colleague viewed as deserving 87%. Although students will have had more stringent and more forgiving teachers, this variation should be random and evenly distributed between those with and without a concussion. In Manitoba, standardized testing in Math and English is offered and passing versus failing is recorded in the Manitoba Education database. However, writing these tests is not mandatory and for this reason, were not part of the analysis.

There may be covariates that predict academic outcomes that were not in the datasets, such as attention deficit hyperactivity disorder, concussion history, specific teachers, learning styles, motivation to do well at school, and attendance habits. With perhaps the exception of attendance and concussion history, these factors should be evenly distributed between the two groups. In particular, future research should conduct subgroup analyses concerning number of concussions and academic outcomes and if those with attention deficit hyperactivity disorder were receiving academic accommodations prior to their concussion. Additionally, the databases did not collect the use of RTL Programs or if a student was provided academic accommodations during their concussion recovery. The lack of difference in adjusted GPAs between the concussed and non-concussed students may be due to concussed students receiving academic accommodations. However, we excluded students whose concussions occurred near the end of the school or semester year to eliminate the potential of teachers using a marks-carried-forward approach. Also, the notion of cognitive rest and RTL was not well known in 2006–2011. The Canadian Pediatrics Society reports on RTL in the 2014 publication [40] of pediatric sport-related concussion but is not included in the 2012 publication [41]. The timing of our included dataset reduces the likelihood of students receiving extensive academic accommodations. Finally, mechanism of injury (e.g., sport-related concussion, motor vehicle collision, falls) is not reported in the databases and we were unable to explore results by injury mechanism. In general, our results are population-based and may not be generalizable to specific concussion mechanisms and additional research is needed.

There was not enough statistical power to conduct a sensitivity analysis among PCS patients. Evidence suggests that some pediatric concussion patients who develop PCS may be at an elevated risk of depression or anxiety [42], vestibulo-ocular dysfunction [43], or chronic migraines [44]. PCS and these associated factors may have adverse effects on academic performance that would be less likely to have affected patients who recovered sooner. PCS has gained increasing awareness in the last several years [45–47] and this condition may have been underreported or under recognized between 2006–2011. It is unclear if our findings can be generalized to students who develop PCS. In addition to examining effect of PCS, other important considerations for future studies should include time loss from school, role of tailored Return-to-Learn programs, effects of multiple concussions, concussion severity, and concussion management on academic outcomes.

## Conclusions

The results of this population-based study suggest that concussion does not appear to have a significant long-term impact on academic outcomes during the high school years. Future studies should examine potential changes in term or report card grades in case there are more immediate, short-term consequences that cannot be observed using each of the year teacher assigned grades. Also, prospective studies are needed to determine risk factors for poor academic performance after concussion, the effect of PCS on academic performance, further explore differences in course specific grades post concussion, and health-related quality of life compared to those without concussion.
